# Diet-dependent modulation of energy balance by CB1 signaling in peripheral sensory neurons

**DOI:** 10.1016/j.isci.2025.113124

**Published:** 2025-07-15

**Authors:** Benjamin Linden, Hussein Herz, Mohammad Jarrah, Dana Tasabehji, Sanaz Saleh, Aviva Fraer, Patrick Clark, Yuanchao Ye, Yi Chu, Zeina Al-Khalil, Donald A. Morgan, Zhiyong Zhu, Carlos M. Castorena, Leonid Zingman, Kamal Rahmouni, Mohamad Mokadem

**Affiliations:** 1 Department of Internal Medicine, Roy J. and Lucille A. Carver College of Medicine, University of Iowa, Iowa City, IA, USA; 2 American University of Beirut, Faculty of Medicine, Beirut, Lebanon; 3 Department of Neuroscience & Pharmacology, Roy J. and Lucille A. Carver College of Medicine, University of Iowa, Iowa City, IA, USA; 4 Division of Hypothalamic Research, Department of Internal Medicine, The University of Texas Southwestern Medical Center, Dallas, TX, USA; 5 Iowa Neuroscience Institute, Roy J. and Lucille A. Carver College of Medicine, University of Iowa, Iowa City, IA, USA; 6 Fraternal Order of Eagles Diabetes Research Center, University of Iowa, Iowa City, IA, USA; 7 Obesity Research & Education Initiative, University of Iowa, Iowa City, IA, USA; 8 Veterans Affairs Health Care System, Iowa City, IA, USA

**Keywords:** molecular neuroscience, cellular neuroscience, sensory neuroscience

## Abstract

The endocannabinoid system plays a pivotal role in metabolic regulation, primarily through cannabinoid receptor-1 (CB1) signaling. In this study, we show that rimonabant (Rim), a selective non-restricted CB1 antagonist, induces substantial weight loss across multiple diet groups, although reduced food intake occurred only in the high-fat (HF) diet group. Rim enhanced brown adipose tissue (BAT) thermogenesis across all diets and visceral white adipose tissue (vWAT) thermogenesis in HF and high-carbohydrate (HC) diets. We identified CB1 expression in the gut’s splanchnic and vagal neurons and found that CB1 modulation significantly influenced afferent splanchnic but not vagal neuronal activity. Furthermore, selective splanchnic, not vagal, afferent denervation eliminated Rim’s anorectic effect. Mice lacking CB1 in sensory neurons (Nav1.8Cre/CB1^flox/flox^) showed reduced diet-induced weight gain and diminished metabolic response to JD5037, a peripherally restricted CB1 antagonist. These findings emphasize the importance of CB1 signaling in sensory neurons as a key mechanism regulating energy homeostasis.

## Highlights

• Rimonabant boosts thermogenesis in BAT and vWAT, with diet-specific energy effects
• CB1 agonist/antagonist modulation alters splanchnic—not vagal—afferents’ activity
• The anorectic effect of CB1 antagonism is mediated through splanchnic afferents
• CB1 receptors in gut sensory neurons regulate food intake and vWAT thermogenesis

## Graphical abstract

**Figure:**
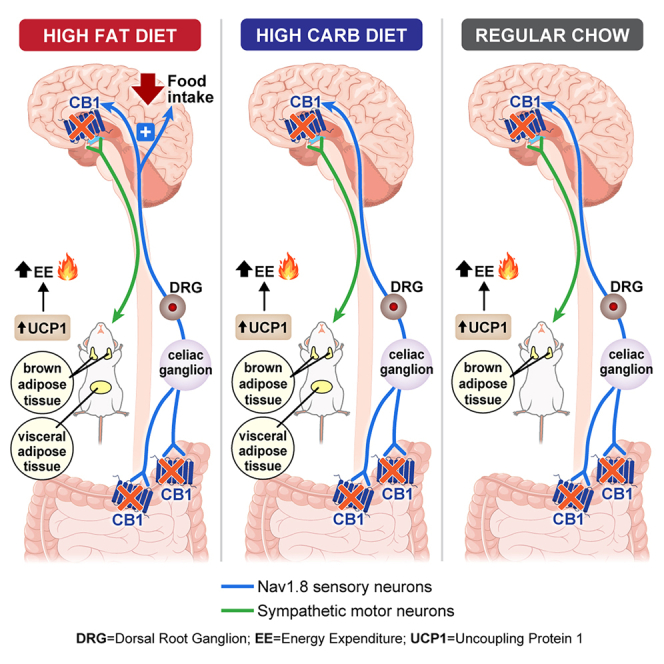

## Introduction

The escalating prevalence of obesity represents one of the most pressing global public health challenges.^1^ Global prevalence of obesity has more than doubled between 1980 and 2015 in over 70 countries with recent estimates revealing that a staggering 603.7 million adults worldwide are affected by this condition.^1^ Alarming statistics indicate that over 40% of the global population is currently classified as overweight or obese.^1^ In the United States, the National Health and Nutrition Examination Survey reported a prevalence of obesity of ∼40% in 2015–2016, and this rate continues to rise.^2^ To comprehend the complex etiology of obesity, it is imperative to understand the multifactorial nature of this condition, encompassing biological and genetic factors, socioeconomics, environmental influences, and behavioral components.^1^ The Western diet, characterized by its high-glycemic/high-insulinemic carbohydrate content and significant fat consumption, has been implicated as a contributing factor.^3^^,^^4^ Extensive research in rodent models has indicated a direct correlation between high-fat (HF) diets and weight gain, excess adiposity, hyperglycemia, insulin resistance, and altered caloric intake and feeding behavior.^5^^,^^6^ Investigations into the underlying mechanisms of excess weight gain, hyperphagia, and altered energy balance have identified the endogenous endocannabinoid system (ECS) as a key component of feeding behavior and energy homeostasis.^7^^,^^8^ Endocannabinoids are small lipid molecules (derived from arachidonic acid) that bind to CB1 and CB2 receptors located in the brain and periphery. In the brain, the highest expression of CB1 is in the GABAergic interneurons, pyramidal neurons, and glutamatergic neurons of the hippocampus, cerebral cortex, and basal ganglia.^9^ In the periphery, CB1 receptors have been reported to be expressed in different organs such as the adrenal glands, adipocytes, peripheral neurons, and the gastrointestinal tract.^10^^,^^11^^,^^12^ However, the exact molecular and cellular mechanisms by which CB1 receptors in the periphery affect energy balance are still not fully understood. Interestingly, tetrahydrocannabinol, the main psychoactive compound in cannabis, has been shown to stimulate appetite and increase food intake predominantly through CB1 receptor activation.^7^^,^^13^ Endocannabinoids share this modulatory effect on feeding behavior.^13^ Intriguingly, mice fed a high-fat (HF) diet exhibit elevated hepatic levels of anandamide (AEA), an endocannabinoid agonist, as well as increased basal rates of fatty acid synthesis compared to those on a normal diet, suggesting that diet composition can differentially modulate the ECS.^14^^,^^15^ Additionally, levels of endocannabinoids, including AEA and 1,2-arachidonylglycerol, are increased in the serum and visceral adipose tissue in obese humans compared to lean counterparts, indicating that the peripheral ECS may be upregulated in obesity.^16^^,^^17^ Male mice treated with AEA during lactation exhibited increased body fat content and CB1 receptor levels in epididymal adipose tissue.^18^ Studies utilizing CB1 receptor knockout mice have demonstrated their resistance to diet-induced obesity (DIO), even though their total caloric intake remains unchanged, indicating the pivotal role of the peripheral endocannabinoids and CB1 receptor in the regulation of energy metabolism.^19^ Previous reports have also revealed the critical involvement of the ECS in the positive feedback mechanism driving fat intake.^20^ Central administration of cannabinoids (into the ventromedial hypothalamus and limbic forebrain) induced significant hyperphagia and increased eating motivation in rodents.^21^^,^^22^^,^^23^ Furthermore, administration of the CB1 receptor inverse agonist rimonabant to mice resulted in transient reductions in food intake, increased energy expenditure, and enhanced thermogenesis in visceral white adipose tissue (vWAT).^10^^,^^24^^,^^25^^,^^26^ To understand how CB1 receptor manipulation influences energy balance, particularly concerning diet composition, we assessed how various diets affect the response evoked by CB1 receptor inverse agonist treatment. We then explored the peripheral localization of CB1 in the gut, specifically in relation to afferent/sensory neurons, and used several physiologic and genetic manipulations of autonomic nerve fibers to examine and delineate the metabolic effects of CB1 activation versus blockade. Our findings shed light on the intricate processes governing energy homeostasis and contribute to our understanding of obesity pathogenesis, potentially paving the way for novel therapeutic interventions.

## Results

### Differential diet-dependent effect of rimonabant on energy balance and adipose tissue thermogenesis

**Figure 1:**
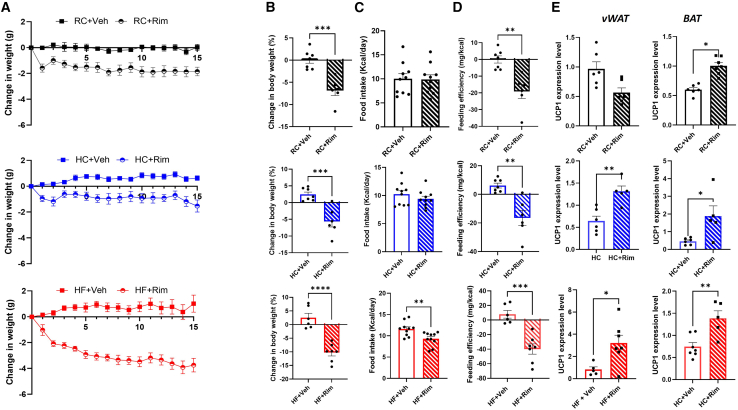
The effect of rimonabant on measurements of energy balance across different diet groups (A) Weight change in (g) over time in (days). (B) Percent (%) change from baseline in total body weight at day 15. (C) Average daily food intake in (Kcal/day) measured during week 1 (over 3 days period). (D) Feeding efficiency displayed in (mg of weight change/Kcal consumed) over 15 days. (E) UCP1 mRNA expression in visceral white adipose tissue (vWAT) and brown adipose tissue (BAT) of C57BL/6J male mice placed on regular chow (RC), high-carbohydrate (HC), and high-fat (HF) diets and given vehicle vs. rimonabant for 15 days. All results are presented as mean ± SEM (error bars). *n* = 5–7. Student’s t test was used to compare means of each diet intervention. Statistical significances are denoted with asterisks as follows: *∗p* ≤ 0.05, *∗∗p* ≤ 0.01, *∗∗∗p* ≤ 0.001; and *∗∗∗∗p* ≤ 0.0001.

**Figure 2:**
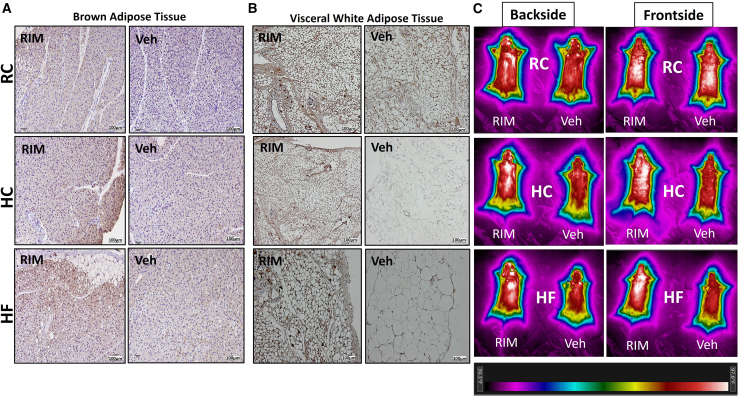
Thermogenic effect of rimonabant on brown and white adipose tissue across different diets (A and B) Immunohistochemical staining of UPC1 (reddish-brown) in (A) brown adipose tissue (BAT) and (B) visceral white adipose tissue (vWAT) of rimonabant vs. vehicle-treated C57BL/6J mice across several diet groups. RC, regular chow HC, high carbohydrates; HF, high fat. Scale bars, 100 μm. (C) *In vivo* recording of body heat temperature—measured by infrared (IR) camera—from the backside (BAT localized region [left]) and the frontside (vWAT localized region [right]) of C57BL/6J mice, 60 min after administration of rimonabant vs. vehicle across several diet groups. The colored heat grade legend is displayed below IR images. *n* = 3–4.

Mice on HF and high-carbohydrate (HC) exhibited a significantly increased average daily caloric intake when compared to mice on regular chow (RC) (Figure S1A). This was associated with significantly higher body weight in mice on HF and HC (Figure S1B). Despite a similar caloric consumption, both the HF and the HC diet groups showed significantly greater total body weight gain compared to the RC group, with the HF group exhibiting a 66.4% increase from baseline and the HC group a 33.8% increase after 8 weeks of feeding (Figure S1C). Similarly, HF mice had a 26% increase in body fat, compared to only 8.9% in HC and 1% in RC groups (Figure S1D). Furthermore, HF mice had a significantly lower average daily heat production compared to the HC and RC groups measured by indirect calorimetry (Figures S1E and S1F). Accordingly, VO_2_, VCO_2_, rate exchange ratio (RER), and fecal calorie loss were lower in the HF group compared to HC and RC groups (Figures S1G–S1I). We next explored differences in thermogenic regulation across several adipose tissues. We found an increase in uncoupling protein 1 (UCP1) expression in BAT of mice on HC compared to those on HF and RC and a decrease in UCP1 within subcutaneous white adipose tissue (scWAT) of HF fed mice compared to RC-fed controls (Figure S1J). Next, we examined the effect of CB1 inverse agonist, rimonabant (Rim), on mice fed the three diets (RC, HC, and HF). All diet groups had a significant decrease in body weight when given Rim for 2 weeks compared to the vehicle control group, which displayed overall no significant change in body weight (Figures 1A and 1B). Interestingly, the HF group demonstrated the greatest decrease in body weight losing about twice as much weight as the other 2 groups. Moreover, the HF group was the only group to show a significant food intake reduction in response to Rim (Figure 1C). The feeding efficiency, measured as weight gained (mg) per calorie consumed (kcal), was significantly decreased in all groups with a more pronounced response in the HF mice (Figure 1D). Examination of the differential effect of Rim on adipose tissue thermogenesis based on diet composition showed that UCP1 mRNA was elevated in BAT of all diet groups and in vWAT of HC and HF diet groups (Figure 1E). This distinct diet-dependent effect of Rim on fat thermogenesis was further reinforced by an increase BAT UCP1 protein expression across all diet groups by immunohistochemistry (IHC) (Figure 2A) and by immunofluorescence (IF) (Figure S2). On the other hand, vWAT UCP1 expression was only enhanced in rimonabant-treated mice on HC and HF but not on RC diets (Figures 2B and S3). Furthermore, *in vivo* assessment of surface body temperature—using an infrared camera—around the BAT area (backside of mouse) and vWAT area (frontside of mouse) in response to Rim validated the aforementioned molecular findings (Figure 2C). Collectively, these data indicate that Rim differentially induces thermogenesis in vWAT of HC and HF mice and in BAT of all diet groups. Rim also induced differential lipolytic activity across various diet groups as ATGL mRNA expression was elevated in vWAT of HC and HF diet groups and in scWAT of HF group only (Figures S4A and S4B). Finally, the selective anorectic effect of Rim in the HF versus the HC group is further supported by a differential effect on major orexigenic and anorexigenic peptides in the hypothalamus where we saw increased hypothalamic neuropeptide Y expression in HC but not HF mice treated with Rim compared to their controls (Figures S4C and S4D). While hypothalamic agouti-related peptide showed an increased tendency toward significance in response to Rim only in the HC, proopiomelanocortin expression was not affected by Rim in either diet group (Figures S4C and S4D).

### CB1 localizes to gut sensory neurons while CB1 modulation selectively alters afferent splanchnic fibers activity

**Figure 3:**
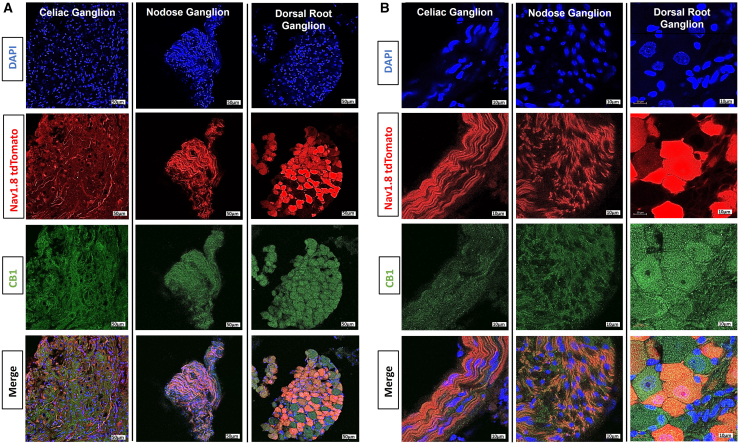
Localization of CB1 receptor within afferent/sensory neurons CB1 immunostaining of cross-sections of the celiac ganglion, nodose ganglion, and thoracic (T10) dorsal root ganglion of Nav.1.8-tdTomato mice (CB1 = green, Nav1.8-tdTomato = red, DAPI = blue. (A) 20×; scale bars, 50 μm and (B) 100×; scale bars, 10 μm. *n* = 4–5 mice.

**Figure 4:**
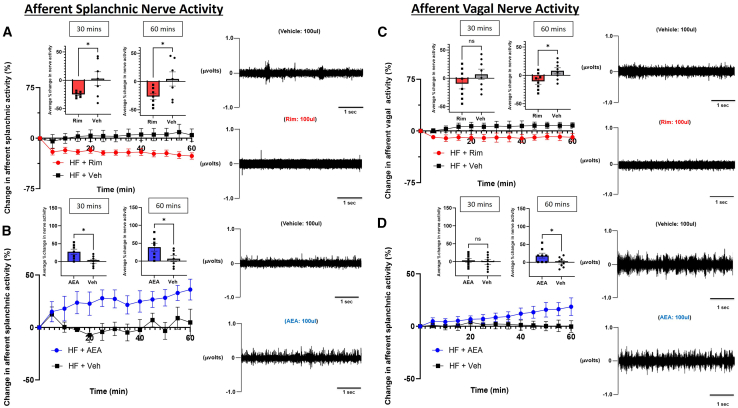
Effects of CB1 agonist/antagonist on direct neuronal activity of splanchnic vs. vagal afferent fibers (A and B) Direct multi-fiber recording of afferent splanchnic nerve activity following administration of (A) rimonabant (Rim) vs. vehicle (Veh) or (B) anandamide (AEA) vs. Veh. (C and D) Direct multi-fiber recording of afferent vagal nerve activity following administration of (C) Rim vs. Veh or (D) AEA vs. Veh. A representative neurogram on the left side corresponds to each intervention at times 30 and 60 min. *n* = 7–8. All results are presented as mean ± SEM (error bars). Student’s t test was used to compare the means of each diet. Statistical significances are denoted with asterisks: *∗p* ≤ 0.05.

Given the importance of the sympathetic nervous system in the control of energy expenditure via induction of UCP1, we sought to identify which neurons mediate the effects evoked by CB1 receptor activation. We first characterized the localization of CB1 receptor in the viscera via immunofluorescence staining. Using small intestinal cross-sections of HF mice, we found strong co-localization of CB1 with markers of sensory/afferent neurons (CGRP) and partial co-localization in chromogranin A expressing cells (enteroendocrine cells) (Figure S5). On the other hand, there was minimal co-localization of CB1 with CD45-expressing (leukocytes) and CD68-expressing (macrophages/monocytes) cells (Figure S6). Using Nav1.8^Cre+^/tdTomato^27^ mice to label sensory neuron fibers, we found high co-localization of CB1 with Nav1.8-positive cells in the celiac ganglion, nodose ganglion, and dorsal root ganglion (Figures 3A and 3B). To elucidate the role of sensory neuron fibers in the gut in mediating peripheral CB1 receptor signaling, we measured splanchnic and vagal afferent nerve activity following direct luminal administration of the CB1 receptor agonist AEA or the inverse agonist Rim in HF mice. Notably, splanchnic afferent nerve activity was significantly decreased in Rim-treated mice compared to vehicle controls, whereas treatment with AEA significantly increased splanchnic nerve activity at both 30- and 60-min post-administration (Figures 4A and 4B). In contrast, vagal afferent nerve activity showed no significant changes at 30 min following either Rim or AEA administration; however, at 60 min, a mild decrease in activity was observed with Rim, and a mild increase was seen with AEA (Figures 4C and 4D). The specificity and accuracy in distinguishing afferent from efferent neuronal fiber activity was validated by intracerebroventricular (ICV) administration of leptin, which selectively increased efferent (sympathetic motor) fiber activity without affecting afferent (sensory) fiber activity (Figure S7). Collectively, these data indicate that peripheral CB1 receptor signaling selectively modulates afferent splanchnic nerve activity.

### Afferent denervation of splanchnic and not vagal nerves attenuates Rim-induced anorectic effects

**Figure 5:**
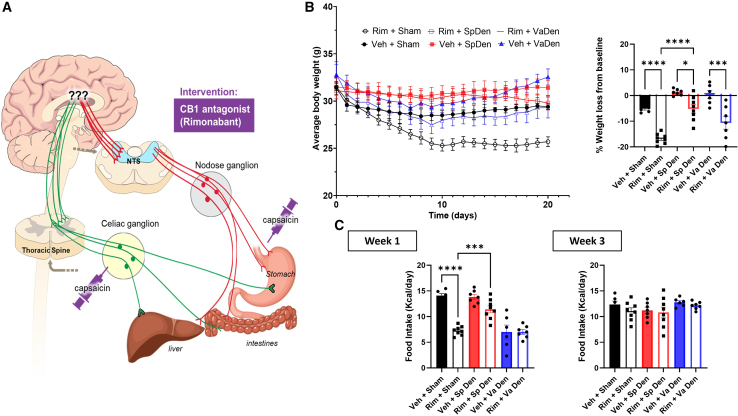
Effects of splanchnic versus vagal sensory denervation on energy balance in response to cb1 antagonism (A) Schematic display of experiments examining the effects of CB1 antagonist (rimonabant) in mice with selective splanchnic vs. vagal afferent denervation. (B) Absolute body weights expressed in grams (g) (left) and percent (%) weight changes from baseline (right) after 20 days of rimonabant vs. vehicle administration in sham denervated, afferent splanchnic denervated, and afferent vagal denervated high-fat diet-induced obese C57BL/6J mice. (C) Average daily food intake expressed in (Kcal/day) during week 1 (left) and week 3 (right) of rimonabant vs. vehicle administration in sham-denervated, afferent splanchnic denervated, and afferent vagal denervated C57BL/6J mice. All results are presented as mean ± SEM (error bars). *n* = 6–8. One-way ANOVA was used to compare means between interventions. Statistical significances are denoted with asterisks as follows: *∗p* ≤ 0.05, *∗∗∗p* ≤ 0.0001, and *∗∗∗∗p* ≤ 0.00001.

To examine the requirement of afferent splanchnic vs. vagal nerves for Rim-induced metabolic effects, we developed two mouse models of selective afferent splanchnic (Figure S8) as well as vagal denervation (Figure S9) as mentioned in the “STAR Methods” section and validated them accordingly. The selective afferent splanchnic denervation was confirmed by near-complete loss of CGRP in the small intestine (jejunum) compared to sham animals (Figure S8A). We further confirmed that the denervation process was specific to sensory neurons, as no effect on tyrosine hydroxylase (TH) expression (motor/sympathetic neurons) was observed (Figure S8B). In addition, to ensure that the selective afferent denervation was selective to the splanchnic/spinal tract and not the vagal afferent pathway, we confirmed by immunofluorescent staining that CGRP expression was mainly decreased in the celiac and not the nodose ganglion (Figures S8C and S8D). Selective afferent vagal denervation was confirmed by showing a lack of CGRP expression in nodose ganglion after 5 days of capsaicin application and no effect on CGRP expression in cervical C3 DRG (Figure S9). Using these two selective denervation models, we sought to examine Rim-induced metabolic effects in mice deficient in either sensory tract (Figure 5A). We observed an 11.7% weight loss difference between Rim and vehicle-treated mice in the HF-fed sham denervation (sham) group compared to a 6.4% difference in splanchnic-denervated and 10.9% in vagal-denervated mice (Figure 5B). The Rim-induced reduction in food intake (anorectic effect) was evident during week 1 and not week 3 of drug administration, as we previously demonstrated.^10^ Furthermore, afferent vagal denervation (Va Den) showed a reduction in food intake during week 1 post-surgery when compared to sham denervation, but splanchnic denervation (Sp Den) did not (Figure 5C). Despite that Rim-induced anorectic effect (during week 1) was absent in both Sp Den and Va Den mice when compared to their vehicle-treated counterparts, Sp Den mice showed higher caloric consumption than Va Den and sham controls in response to Rim (Figure 5C). These data suggest that the anorectic effect of Rim is mediated by CB1 receptors on afferent spinal neurons and is diet-composition dependent.

### CB1 deletion in sensory neurons eliminates the anorectic effect of CB1 antagonism

**Figure 6:**
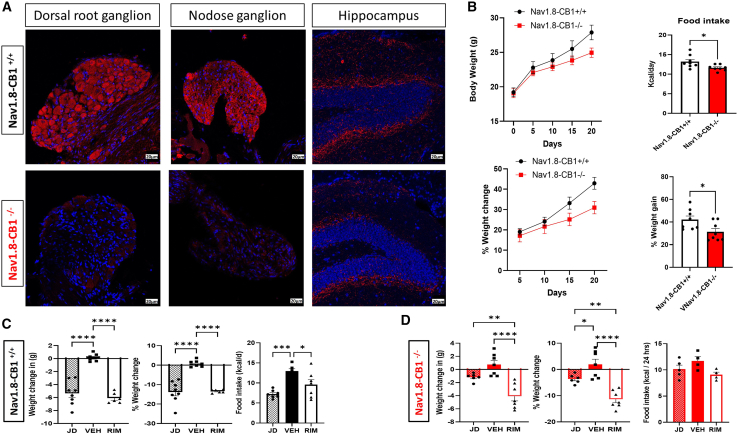
CB1 deletion in sensory neurons eliminates the anorectic effect of cb1 antagonism (A) CB1 immunostaining (red) in thoracic-T10 dorsal root ganglion (DRG), nodose ganglion, and hippocampus of 12-week-old male Nav1.8-Cre^+^/CB1^flox/flox^ (Nav1.8-CB1^+/+^) and Nav1.8-Cre^−^/CB1f^lox/flox^ (Nav1.8-CB1^−/−^) mice. Scale bars, 20 μm. (B) Body weight in (g) over time, average daily food intake in (Kcal/day), % weight change from baseline over time, and % weight gain after 3 weeks (21 days) of HF diet feeding in Nav1.8-CB1^+/+^ and Nav1.8-CB1^−/−^ male mice. (C and D) Body weight changes (expressed in grams [g] and percent [%]) and average daily food intake (measured over 3 days during week 1) in HF diet-fed (C) Nav1.8-CB1^+/+^ and (D) Nav1.8-CB1^−/−^ male mice after 15 days of administration of peripherally restricted CB1 antagonist (JD5037) vs. non-restricted CB1 antagonist (Rim) vs. vehicle (Veh). *n* = 6–8. All results are presented as mean ± SEM (error bars). Student’s t test was used to compare means between two groups, and one-way ANOVA was used to compare means between interventions. Statistical significances are denoted with asterisks as follows: *∗p* ≤ 0.05, *∗∗p* ≤ 0.01, *∗∗∗p* ≤ 0.0001, and *∗∗∗∗p* ≤ 0.00001.

**Figure 7:**
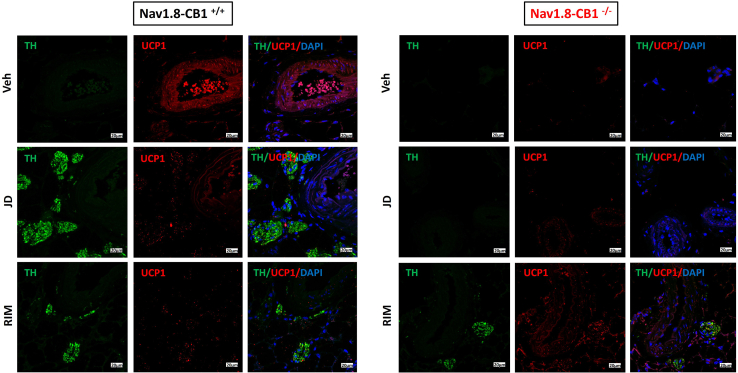
CB1 deletion in sensory neurons eliminates the thermogenic effect of CB1 antagonism in visceral WAT TH and UCP1 co-expression—assessed by immunofluorescence (IF) in HF diet-fed Nav1.8-CB1^+/+^ and Nav1.8-CB1^−/−^ male mice after 15 days of administration of peripherally restricted CB1 antagonist (JD) vs. non-restricted CB1 antagonist (Rim) vs. vehicle (Veh). *n* = 3–4. DAPI = blue, TH = green, UCP1 = red. Scale bars, 20 μm.

To further explore the role of CB1 signaling in spinal (splanchnic) neurons on energy balance, we generated a transgenic mouse model of sensory neuron-specific deletion of CB1 (Nav1.8^Cre+^/CB1^flox/flox^) by crossing Nav1.8^Cre+^ mice with CB1^flox/flox^ mice. We confirmed CB1 deletion in both vagal sensory neurons (nodose ganglia) and spinal sensory neurons (dorsal root ganglia and celiac ganglia) but not in the central nervous system (hippocampus) (Figure 6A). Characterization of these Nav1.8^Cre+^/CB1^flox/flox^ (Nav1.8-CB1^−/−^) mice in comparison to their littermate controls Nav1.8^Cre−^/CB1^flox/flox^ (Nav1.8-CB1^+/+^) showed that they have a diminished weight gain and reduced food intake in response to HF diet challenge (Figure 6B). More importantly, these mice have a significantly attenuated weight loss response and an absent anorectic effect to either Rim (a non-restricted CB1 antagonist) or JD5037 (a peripherally restricted CB1 antagonist) (Figures 6C and 6D). JD5037 was still able to induce a sympathetic-mediated thermogenic response in vWAT of Nav1.8^Cre−^/CB1^flox/flox^ (Nav1.8-CB1^+/+^) but that effect was lost in the Nav1.8^Cre+^/CB1^flox/flox^ (Nav1.8-CB1^−/−^) (Figure 7).

## Discussion

The effective management of obesity remains a difficult challenge for global public health, particularly in the context of the pervasive Western diet, which fosters an obesogenic environment. This study aimed to elucidate the differential impacts of HF and HC diets on energy balance and the role of CB1 signaling in these processes. Our findings reveal significant insights into how these diets differentially affect caloric intake, body weight, fat mass, and energy expenditure, as well as the thermogenic responses of various adipose tissues. Our results indicate that both HF and HC diets lead to significantly higher caloric intake compared to RC. Despite similar caloric consumption, HF-fed mice exhibited more pronounced weight gain and increased fat mass compared to HC-fed mice. This discrepancy underscores the differential metabolic handling of dietary fats versus carbohydrates. Notably, HF-fed mice showed reduced energy expenditure, as evidenced by lower heat production and a decrease in UCP1 expression within scWAT, which aligns with previous studies highlighting the obesogenic potential of HF diets through enhanced post-ingestive caloric availability and diminished thermogenesis.^28^ Our study demonstrates that administration of the CB1 inverse agonist Rim induces significant weight loss across all diet groups, with the most pronounced effect observed in HF-fed mice. This weight loss was accompanied by a substantial reduction in daily food intake and increased feeding efficiency, suggesting that Rim’s effects are mediated through both suppression of caloric intake and enhancement of energy expenditure. These findings are consistent with previous reports indicating that CB1 receptor antagonism can modulate metabolic disorders and promote weight loss by affecting both food intake and energy balance.^29^^,^^30^^,^^31^^,^^32^^,^^33^^,^^34^ Rim administration promoted thermogenesis and fat browning across different adipose tissues. UCP1 expression and heat production were elevated in vWAT of HF- and HC-fed mice in response to Rim compared to their controls, but not RC-fed mice. Additionally, Rim increased UCP1 expression and measured heat production in BAT of all diet groups, suggesting diet-dependent modulation of thermogenic pathways. The feeding efficiency following Rim was significantly decreased in all diet groups, suggesting an increase in energy expenditure, but the effect was most pronounced in the HF mice. Collectively, these findings show that Rim induces BAT thermogenesis across all diet groups but selectively induces vWAT browning in mice fed obesogenic diet (HC and HF). The observed increase in lipolysis markers (ATGL) within vWAT of HC and HF mice further supports the notion that Rim differentially enhances lipolysis and thermogenesis based on diet composition, a finding corroborated by previous studies.^35^^,^^36^ Understanding the communication between gut endocannabinoids and the central nervous system in regulating food intake is crucial. Our data indicate that CB1 receptors in the gut play a significant role in mediating the anorectic effects of Rim, particularly through afferent splanchnic neurons. We previously showed that the effect of Rim on food intake reduction (but not energy expenditure or fat thermogenesis) was eliminated after splanchnic denervation in diet-induced obese mice, suggesting that peripheral CB1 receptors in the gut’s nervous system might be responsible for some of the anorectic effects of Rim.^10^ Our recent study elucidates the localization of peripheral CB1 receptors to sensory neurons within intestinal tissues, showing an abundant CB1 expression within the celiac ganglia, dorsal root ganglia, and the nodose ganglia. The contrasting nerve activity response to CB1 agonists and antagonists highlights the selective modulation of splanchnic nerve activity by CB1 signaling, which could be an important mechanism in the regulation of energy balance. We then demonstrated that afferent vagal denervation had minimal effect on weight loss and energy balance outcomes in Rim-treated HF-fed mice. On the other hand, afferent splanchnic denervation took away the early food reduction effects of Rim in the same HF group. This selective effect of peripheral CB1 antagonism on energy balance through splanchnic afferent fibers rather than vagal afferent fibers is further supported by one previous study showing that CB1 deletion in vagal neurons (using Phox2b^Cre^/CB1^flox/flox^ mice) did not have any consequences on body weight or food intake^27^ contrary to our Nav1.8^Cre+^/CB1^flox/flox^ mouse model that demonstrated decrease in food intake and resistance to weight gain on HF diet. Furthermore, the loss of Rim-induced anorectic effect in these mice with pan-sensory deletion of CB1 highlights the importance of peripheral CB1 in the regulation of food intake. Our results reveal that peripheral CB1 antagonism affects energy balance, specifically food intake, through Nav1.8-expressing splanchnic (spinal) rather than vagal sensory neurons. Our findings are further reinforced by a previous study showing that peripheral CB1 antagonism via spinal sensory neurons induces hypophagia through a leptin-melanocortin-dependent pathway in the hypothalamus.^34^ Moreover, another study highlighted a significant role of Nav1.8 expressing neurons in communicating energy signals across the gut-brain axis by regulating periodic food intake and microbial interaction with the host.^37^ The Nav1.8-Cre mouse model, used as a specific sensory neuron marker, shows minimal alterations in neuronal functions when crossed with other p-loxed strains.^38^^,^^39^ Although several studies have shown that vagal afferent fibers are essential for regulating other meal termination and reward-related feeding behaviors,^40^^,^^41^^,^^42^ our findings suggest that spinal sensory pathways mediate the selective effects of peripheral CB1 signaling on food intake, possibly by affecting other feeding behavior aspects such as meal initiation or interval periods between meals. This adds to the growing evidence that multiple, distinct neural circuits contribute to the complex control of feeding and energy homeostasis.

Interestingly, restricted CB1 antagonism (using JD5037 compound) induced a sensory/sympathetic reflex to visceral fat to promote thermogenesis only in wild-type Nav1.8-CB1^+/+^ animals, while Rim maintained its effect on sympathetic-mediated vWAT thermogenesis in Nav1.8- CB1^−/−^ mice. These findings suggest that CB1 activation of a sympathetic-mediated loop to induce browning of visceral fat occurs peripherally and centrally. Although another study showed that celiac/sympathectomy or subdiaphragmatic vagotomy did not alter the effects of rimonabant on energy balance in rats,^43^ these findings were confounded by the fact that this study’s vagotomy and sympathectomy models did not affect weight or food intake, contrary to what was shown in previous reports^10^^,^^44^^,^^45^^,^^46^. In that same study, Rim induced a reduction in food intake of RC-fed rats, a result that was observed in our mouse model only on the HF diet. This raises questions about potential interspecies differences in homeostatic energy regulation. The observation that modulation of energy balance by CB1 inverse agonists is influenced by diet highlights the potential role of gut microbiota in modulating cannabinoid pathways along the gut-brain axis.

In conclusion, our study elucidates the complex interplay between diet composition, CB1 signaling, and gut microbiota in the control of energy balance. These findings provide further insights into the mechanisms by which endocannabinoids influence feeding behavior and metabolic health, highlighting the potential for targeted therapies that modulate CB1 signaling to manage obesity. Future research should further explore these interactions and their suitability as effective strategies for managing obesity and related metabolic disorders.

### Limitations of the study

This study primarily utilized male murine models to investigate CB1 signaling, which, while informative, may not fully capture the complexity of metabolic regulation across genders. Additionally, although diet-dependent responses were observed, only three dietary compositions were tested, limiting the generalizability across broader nutritional contexts. Finally, the effects of chronic CB1 modulation beyond the short treatment windows remain unclear. Future studies incorporating human-derived tissues or translational models, longer treatment durations, and a wider range of dietary profiles, including sex as a biological variable, will be important to better understand the therapeutic potential and limitations of peripheral CB1 signaling in metabolic regulation.

## Resource availability

### Lead contact

Further information and requests for resources and reagents should be directed to and will be fulfilled by the lead contact, Mohamad Mokadem (mohamad-mokadem@uiowa.edu).

### Materials availability

This study did not generate new or unique reagents.

### Data and code availability

All original code is available in this paper as well as in its supplemental information. Any additional information required to reanalyze the data reported in this paper is available from the lead contact upon request.

## Acknowledgments

We would like to thank Dr. Joel Elmquist (UT Southwestern Medical Center, Dallas, TX) for his support and for providing a key transgenic mouse model for our studies. We would also like to thank the Metabolic Phenotyping Core Facility at the University of Iowa for measurements of energy expenditure using the CLAMS and body composition assessment using Nuclear Magnetic Resonance (NMR). Finally, we would like to thank Teresa Ruggle (Senior Designer/Art Director in the internal Medicine department at the University of Iowa) for her assistance in designing schematic figures and the visual abstract.

M.M. is supported by funds from the US Department of Veterans Affairs, VA Merit Review Program (BX004774-05), the Department of Internal Medicine at the University of Iowa Carver College of Medicine, and the University of Iowa Fraternal Order of Eagles Diabetes Research Center. L.Z. is supported by funds from 10.13039/100000002NIH (R01 HL168752) and the VA (BX004840). K.R. is supported by funds from 10.13039/100000002NIH (R01 HL162773 and R01HL17294), VA (BX004249 and BX006040), and the University of Iowa Fraternal Order of Eagles Diabetes Research Center.

## Author contributions

Conceptualization, B.L., H.H., M.J., and M.M.; formal analysis, B.L., H.H., M.J., D.T., S.S., A.F., P.C., Y.Y., Y.C., Z.A.-K., D.A.M., and Z.Z.; investigation, B.L., H.H., M.J., D.T., S.S., A.F., P.C., Y.Y., Y.C., Z.A.-K., D.A.M., Z.Z., C.M.C., K.R., and M.M.; resources, C.M.C., L.Z., K.R., and M.M.; writing, B.L., H.H., M.J., and M.M.; critical revision, K.R. and M.M.; funding, L.Z., K.R., and M.M.

## Declaration of interests

C.M.C. is currently a Principal Scientist (for Integrated Physiology Research and Global Drug Discovery) employed by Novonorsdik. His contribution to this work was by generating the CB1^flox/flox^ mouse (which was used in the reported studies) at UT Southwestern Medical Center.

## STAR★Methods

### Key resources table

**Table:** 

REAGENT or RESOURCE	SOURCE	IDENTIFIER
**Antibodies**		

Rabbit anti-UCP1 1:100	ABclonal	A5857
Rabbit anti-CB1 1:250	Abcam	ab23703
Rat anti-mouse CD68 1:50	Bio-Rad	MCA1957GA
Rat anti-CD45 1:50	Novus	NB100-77417
Rabbit anti-TH 1:200	Phosphosolutions	#2025-THRAB
Rabbit anti-CGRP 1:1000	Sigma	C8198
Rabbit anti-chromogranin A	Proteintech	10529-1-AP
Alexa 488 donkey anti-rabbit IgG 1:1000	Invitrogen	A32790
Alexa 555 goat anti-rabbit IgG 1:1000	Invitrogen	A21428
Alexa Fluor 488 donkey anti-rat IgG 1:100 1:1000	Invitrogen	A21208

**Oligonucleotides**		

Uncoupling protein 1 (Ucp1, FAM dye)	IDT	Mm.PT.58.7088262Beta
Beta-3 adrenergic receptor (Adrb3, FAM)	IDT	Mm.PT.58.31423945
Adipose triglyceride lipase (Atgl, FAM)	IDT	Mm.PT.56a.13182140
Agouti-related peptide (Agrp, FAM)	IDT	Mm.PT.58.45969609.g
Neuropeptide Y (Npy, FAM)	IDT	Mm.PT.58.29444574
Proopiomelanocortin (Pomc, FAM)	IDT	Mm.PT.58.5917321
Beta-actin (Actb, HEX dye)	IDT	Mm.PT.39a.22214843

### Experimental model and study participant details

#### Mouse care and generation of sensory neuron-specific CB1 null mice

All animal care and procedures were approved by the University of Iowa Animal Care and Use Committee. C57BL/6J (strain #000664), Nav1.8^Cre^ (strain #036564), and Stop^flox/flox^-tdTomato reporter (strain #007914) mice were purchased from the Jackson Lab. CB1^flox/flox^ mice were obtained from Dr. Joel Elmquist’s lab at University of Texas Southwestern Medical Center.^32^ Homozygous male Nav1.8^Cre^ and female CB1^flox/flox^ or Stop^flox/flox^-tdTomato were bred to generate Nav1.8^Cre+^/CB1^flox/+^ or Nav1.8^Cre+^/tdTomato (which was used for CB1 colocalization immunofluorescence with tdTomato) F1. The first generation of male Nav1.8^Cre+^/CB1^flox/+^ mice (F1) were further bred with female CB1^flox/flox^ to produce Nav1.8^Cre+^/CB1^flox/flox^ (sensory neuron-targeted CB1 knockout) and Nav1.8^Cre−^/CB1^flox/flox^ (control) mice, which were used for the experiments described below in study design 3.

### Method details

#### Study design 1

Six-week-old male C57BL/6J mice were allowed to acclimate to single-housing for two weeks. The mice were divided into three diet groups: HF diet (HF) (60% calories from fat D12492, Research Diets), HC diet (HC) (70% calories from carbohydrates (17% sucrose), D12450H, Research Diets), and RC (59% carbohydrates, 23% protein, 18% fat). Body weight and composition were measured using Nuclear Magnetic Resonance (NMR) LF50 in the Metabolic Phenotyping Core Facility at the University of Iowa. Mice were assigned to their respective diets’ *ad libitum* for 7 weeks. Body weight was recorded weekly in all mice that were single-housed to appropriately measure all elements of energy balance.

#### Metabolic phenotyping

After 6 weeks on different diets, daily food intake, activity, and energy expenditure were determined using the Comprehensive Lab Animal Monitoring System that relies on indirect calorimetry (respirometry) to assess energy balance at University of Iowa Metabolic Phenotyping Core Facility. Animals were individually housed for 5 days in metabolic cages with free access to food and water. Data were continuously recorded for 24 h including circadian food intake, O_2_ consumption, CO_2_ production and locomotor activity.

#### Study design 2

Another group of six-week-old male C57BL/6J mice were allowed to acclimate for two weeks. The mice were divided into three diet groups: HF diet HC diet, or RC. Mice were single-housed and fed respective diets for 6 weeks to create the DIO phenotype. A baseline body weight and body composition were measured (body composition through LF50 NMR). Each diet group was then divided into two groups that received equal volumes of vehicle (0.2% Tween 80) or Rimonabant (SR 141716A in 0.2% Tween 80) (Cat#9000484, Cayman Chemical Co.) by oral gavage at a daily dose of 10 mg/Kg for a total of 3 weeks. Body weight was measured daily during gavage period. Daily food intake was measured during week 2. Body composition by NMR at the time before sacrifice was collected, and then the mice were sacrificed after the third week. Several tissues, including BAT, mesenteric WAT, vWAT, and scWAT, were collected. Molecular analysis was performed on adipose tissue to further assess and compare browning, sympathetic activation, and lipolysis among the groups via RT-qPCR as described below. To mark sensory Nav1.8-expressing neurons with tdTomato, male Nav1.8-Cre mice were bred with female Stop-tdTomato reporter mice to produce Nav1.8-tdTomato mice. Ganglia were dissected and immunofluorescently stained for CB1 as described below. CB1 staining (wavelength 488, green) and tdTomato (wavelength 555, red) were imaged together with nuclear DAPI (blue).

#### RT-qPCR

All mice were sacrificed at the conclusion of the experiments, and tissues were collected for molecular analysis via Quantitative Reverse Transcription-Real-Time Polymerase Chain Reaction (RT-qPCR). Total RNA was extracted from the fat tissues (BAT and WAT, including subcutaneous (sc)WAT, and visceral (vWAT)) with TriZol (Thermo Fisher, USA) and RNeasy Mini Kit (QIAGEN, Germany) according to the manufacturers’ instructions. Total RNA was reverse transcribed to cDNA using cDNA High-Capacity Reverse Transcription Kit (Applied Biosystems, Thermo Fisher). cDNA was used for qPCR using PrimeTime gene expression master mix (2x) (1055771, IDT) on a QuantStudio 3(Applied Biosystems, USA). All the information regarding the TaqMan primers/probes that were used are listed in key resources table. Actb was used as a reference gene (with HEX dye), and the gene of interest (FAM dye) was quantified in a single reaction with identical cDNA input. Relative expression levels were determined using the 2^−ΔΔCt^ method.

#### Study design 3

##### Measurement of splanchnic and vagal afferent nerve fiber activity

Six-week-old male C57BL/6J mice were placed on HF diet for 10 weeks before being anesthetized with a ketamine (91 mg/kg BW; IP) and xylazine (9.1 mg/kg BW; IP) mixture. Each mouse was then intubated with a PE-50 to provide an unimpeded airway for the mouse to spontaneously breathe O_2_-enriched room air. Next, a tapered micro-renathane tubing (MRE-40, Braintree Scientific) was inserted into the right jugular vein for infusion of the sustaining anesthetic agent, α-chloralose (initial dose: 12 mg/kg, then a sustaining dose of 6 mg/kg per h). Another tapered MRE-40 catheter was inserted into the left carotid artery for continuous measurement of arterial pressure and heart rate. Core body temperature was monitored with a use of a rectal probe and continuously maintained at 37.5°C by a temperature controller (Physitemp Model MCAT2) that periodically powers on or off a heated surgical lamp to achieve isothermal conditions. Two separate cohorts of mice were prepared for sympathetic or vagal afferent nerve measurements. *Splanchnic Nerve Measurement*: One cohort of mice was equipped for direct multifiber nerve activity recording by carefully isolating a 3–4 mm section of splanchnic nerve proximal to the celiac ganglion in the upper left retro-peritoneal cavity. A bipolar platinum-iridium electrode (36-gauge, A-M Systems) was suspended under the most distal section of the nerve and secured with a limited amount of silicone gel (50–75 μL, Kwik-Sil, WPI) to fully encase the electrode/nerve connection. The silicone gel was allowed to solidify (10–15 min) to completely adhere the nerve to the surface of the electrode hooks. The electrode was then attached to a high-impedance probe (HIP-511, Grass Instruments), and the nerve signal was amplified at 10^5^ times and filtered at 100-Hz and 1,000-Hz cutoffs with a Grass P5 AC pre-amplifier. The amplified and filtered nerve signal was routed to a speaker system and to an oscilloscope (model 54501A, Hewlett-Packard) to monitor the audio and visual quality of the intact splanchnic sympathetic nerve recordings for quantification purposes. The nerve signal was also directed to a resetting voltage integrator (University of Iowa Bioengineering, model B600c) to analyze the total activity (integrated voltage) and finally to a MacLab analog-digital converter (AD Instruments, Castle Hill, New South Wales, Australia, Model 8S) containing the software (MacLab Chart Pro; Version 7.0) that utilizes a cursor to count the number of spikes/second that exceed the background noise threshold. Under a stable plane of anesthesia and normal core body temperature (37.5°C), a continuous recording of basal intact splanchnic sympathetic nerve activity (SpSNA) was performed for 10-15-min to determine baseline total SpSNA (afferent and efferent activity). Next, the most proximal section of the recorded SpSNA fiber was located and carefully sectioned to record afferent SpNA. Next, an intestinal injection of vehicle (100 μL, 0.2% Tween 80 for Rimonabant, sunflower oil for AEA) was slowly performed (over a 20–30 s period) directly into the lumen of the duodenum. The duodenum was coated with liquid Vaseline to protect the intestine while outside of the visceral cavity. The effects of vehicle were measured until afferent SpSNA returned to baseline (approx. 60 min). Using the same exposed duodenum but at a different site, a second intestinal injection of Rimonabant (Rim, 100 μL at 1 mg/mL) or Anandamide (AEA, 100 μL at 2 mg/mL) was made, and 60–70 min were allowed for the effects of Rim or AEA on afferent SpSNA to eventually return to baseline. At the conclusion of each study, mice were euthanized (ketamine 270 mg/kg IV) and tissues harvested for further experiments. *Vagal Afferent Nerve Measurement:* Another cohort of mice was equipped for direct multifiber vagal nerve activity (VgNA) recording by carefully isolating a 2–3 mm length of a right truncal vagal nerve located on the right lateral surface the esophagus. The preparation for VgNA measurement was performed in the same manner as for the SpSNA described above. Under a stable plane of anesthesia and core body temperature (37.5°C), a continuous recording of an intact baseline VgNA (containing both afferent and efferent activity) was measured over a 10-15-min period. The most proximal section of the recorded VgNA fiber was then located and carefully sectioned to allow only the afferent VgNA to be measured. An abdominal incision was made to expose a small section of the duodenum distal to stomach. Intestinal injections of vehicle (100 μL), Rim (100 μL) and AEA (100 μL) were injected as described above. The effects of vehicle, Rim and AEA on VgNA were measured until afferent activity returned to baseline (approx. 60–70 min). At the conclusion of each study, mice were euthanized (ketamine 270 mg/kg IV) and tissues harvested for further experiments. *Validation Experiment for Afferent vs. Efferent neuron measurements*: In a small cohort of 3 male C57 mice; 14 weeks of age, were anesthetized with isoflurane and an intracerebral ventricular cannula implanted into the lateral ventricle. After 10 days of recovery, mice were prepared for *Splanchnic Nerve Measurement* as described above*.* Basal SpSNA was separately recorded in all 3 mice for 20 min. Sham denervation was performed from the recorded splanchnic sympathetic nerve in one mouse. In the second mouse, the splanchnic nerve was sectioned (1-2mm) distal to the recording site for efferent SpNA measurement. In the third mouse, the splanchnic nerve was severed (1–2 mm) proximal to the recording site so that afferent SpNA would be recorded. After allowing 15 min for a stable control period, a single ICV injection of Leptin (5ug) was administered and the responses of the intact, efferent, and afferent SpSNA to the effects of leptin in the 3 separate mice were recorded for the next 4 h. At the end of the study, mice were euthanized (ketamine, 270 mg/kg, IV).

#### Model of afferent splanchnic denervation

Mice were anesthetized and appropriate analgesia administered as described previously.^10^ Selective splanchnic “de-afferenation” was performed by delicately applying 0.2% capsaicin solution or vehicle (oil, sham control group) over the celiac ganglia and all post-synaptic neuronal fibers extending into the small intestinal gut using fine microfiber tips under direct magnified visualization by Zeiss OPMI VISU 200 surgical microscope. We confirmed the efficacy of this strategy in causing afferent splanchnic denervation using different approaches, including immunostaining for calcitonin gene-related peptide (CGRP) in the small intestine, and by examining CGRP and TH expression by western blot within the small intestine. For western blot, tissues were homogenized in tissue lysis buffer (50 mM HEPES pH 7.5, 150 mM NaCl, 1 mM MgCl2, 1 mM CaCl2, 10 mM NaF, 5 mM EDTA, 1% Triton X-100, 2 mM sodium orthovanadate, and Roche cocktail protease inhibitor tablet). Protein samples (30 μg) were injected into 8% SDS-PAGE gel or 15% SDS-PAGE gel for CGRP and electro-transferred to a PVDF membrane (Bio-Rad). After blocking with 5% w/v non-fat dry milk in TBST (0.1% Tween 20), each membrane was probed with anti-TH (2025-THRAB, 1:2,000, PhosphSolutions, Aurora, CO), CGRP (C-8198, 1:2,000, Sigma), or β-actin (60008-1-Ig, 1:100,000, Proteintech) overnight at 4°C, followed with a secondary anti-rabbit or anti-mouse antibody (1:10,000) for TH, CGRP, or β-actin, respectively, at room temperature for 2 h. Visualization was performed with an enhanced chemiluminescence (ECL) detection kit (GE Healthcare, Little Chalfont, UK) followed by. The density of bands was measured using ImageJ.

#### Model of afferent vagal denervation

Mice were anesthetized and appropriate analgesia was administered as described previously.^10^ A midline incision was made from the mid-abdomen to the xyphoid process. The stomach was exteriorized, and the liver lobes retracted to reveal the esophagus extending from the diaphragm to the stomach fundus. Selective vagal “de-afferenation” was performed by delicately applying 0.4% capsaicin solution or vehicle (oil, sham control group) to the entire length of the esophagus where the vagal nerves are embedded on the exterior ventral and dorsal surface on the esophagus. Upon completion, all internal organs were returned to their normal position in the abdominal cavity. The midline incision was sealed first with 4-0 Vycril suture for the abdominal muscle layer and secondly with wound clips for the skin incision. Vagal Afferent Denervated mice were allowed up to 6 weeks for a full recovery. We confirmed the efficacy of this strategy to cause afferent vagal denervation using immunostaining for calcitonin gene-related peptide (CGRP) in the nodose and celiac ganglions for comparison.

#### Rimonabant effects in diet-induced obesity mice with selective afferent splanchnic vs. vagal denervation

Another group of six-week-old male C57BL/6J mice was fed HF diet for 6 weeks and divided into 3 groups: afferent splanchnic denervation, afferent vagal denervation, and sham denervation as described above. Mice were then allowed 48 h of recovery before receiving oral gavage of vehicle (0.2% Tween 80) or Rimonabant (SR 141716A in 0.2% Tween 80) (9000484, Cayman Chemical) at a dose of 10 mg/Kg for 3 weeks. Daily body weight and food intake were tracked during the gavage period.

#### Effect of restricted vs. non-restricted CB1 antagonist in mice deficient of CB1 within sensory neurons

Nav1.8^Cre+^/CB1^flox/flox^ and Nav1.8^Cre−^/CB1^flox/flox^ were started on HF diet starting at 6 weeks of age for 6 weeks before dividing each group into three subgroups, receiving the non-selective CB1 inverse agonist Rimonabant (SR 141716A) at a dose of 10 mg/Kg/day, or the peripheral-restricted CB1 inverse agonist JD5037 (MedChem Express) at a dose of 3 mg/Kg/day,^27^^,^^28^ or vehicle of equivalent volume by oral gavage for 15 days. Weight and food intake were recorded daily.

#### Immunofluorescent (IF) staining

Tissues were harvested, fixed in 4% paraformaldehyde (PFA) for 24 h, and changed to 30% sucrose in PBS for 24 h. Tissues then were embedded in OCT and stored at −80°C. For vWAT, 10 μm cryosections were obtained on Super frost Plus slides (Fisher) using a Leica CM3050S cryostat (OT, object temperature, −30°C, CT, cabinet temperature, −20°C). For all other tissues, 10 μm cryosections were obtained on Super frost Plus slides using a CryoStar NX50 cryostat at −20°C. For IF, slides containing sections (from −80°C) were let dry at room temperature for 60 min, and rinsed in PBS briefly for three changes to remove OCT. Sections were circled with a PAP pen, post-fixed with 4% PFA for 15 min. After rinsing in PBS to remove PFA, sections were blocked with a blocking buffer (5% normal goat or donkey, corresponding to the secondary antibody, serum, and 0.1% Triton X-100 in PBS) for 1 h at room temperature. Primary antibody (see Antibody List for details) in blocking buffer was applied to sections (normal rabbit or rat, corresponding to the primary antibody IgG at 10 μg/ml as negative control) and incubated for 16–20 h at 4°C. Slides were rinsed in PBS for 5 min each for 3 changes. Secondary Goat anti-rabbit IgG Alexa Fluor Plus 555, donkey anti-rabbit IgG Alexa Fluor Plus 488, or donkey anti-rat IgG Alexa Fluor 488 (2 μg/mL) were applied to sections and incubated for 3 h at room temperature. Slides were rinsed in PBS for 5 min each for 3 changes. Sections were then incubated with a nuclear stain VECTASHIELD DAPI mounting medium (Vector Lab) for overnight in a 4°C refrigerator. Slides were mounted with coverslips and sealed with nail polish. For co-staining of CB-1 (rabbit antibody) with the other antigen whose antibody was of also rabbit, a subsequential staining was applied each as described above. Images were taken using a Zeiss 880 confocal microscope. Refer to key resources table for all antibodies used in immunostaining.

#### Immunohistochemical (IHC) staining

Fresh tissues were fixed in 4% paraformaldehyde for 24 h, and then changed to 70% ethanol. Tissues were embedded in paraffin according to standard procedures. Sections 5-μm thick were cut and laid onto slides. Sections on slides were deparaffinized and rehydrated according to standard steps in xylene and successive ethanol. Antigen retrieval was performed in 10 mM NaCitrate buffer (pH6.0), at 95°C for 10 min and slowly cooling down to room temperature. Slides were rinsed in ddH_2_O for 5 min. Sections were incubated with 3% H_2_O_2_ (in methanol) for 10 min to eliminate endogenous peroxidase activity. After rinsing in dH_2_O, sections were blocked with 5% horse serum in PBS-0.1% Triton X-100 at room temperature for 1 h. Sections were then incubated with anti-UCP1 (rabbit, ABclonal A5857; 1:50) or negative control of 1 μg/mL normal rabbit IgG at 4°C for overnight. Slides were washed 3 times in PBS for 3 min each. Anti-rabbit-polymer-HRP (Vector) were applied onto sections and incubated at room temperature for 30 min. Slides were rinsed in PBS 3 times for 3 min each. DAB (Vector) was added to sections and incubated for approximately 3 min until a brown color appeared on sections with primary antibody, while colorless on negative control sections. Slides were dipped in dH_2_O, and subsequently in hematoxylin for 5 s. After rinsing in dH2O, slides were dehydrated in successive ethanol and xylene, and Permount-mounted with coverslips. Images were taken with an Olympus light microscope.

#### Surface body temperature measurement by infrared camera recorder

The body surface temperature of the mice was imaged by using a high-resolution infrared camera (A655sc Thermal Imager; FLIR Systems, Wilsonville, OR). Quantitative analysis of infrared images was performed using FLIR Research IR software version 3.4.13039.1003. Mice were anesthetized with isoflurane (1%). Back and front side surface body temperatures were recorded in the 3 diet groups (RC, HC, and HF) at 0, 15, and 60 min after oral administration of Rimonabant (10 mg/Kg) vs. vehicle (0.2% Tween 80). The side-by-side imaging of mice in the same camera frame allows simultaneous assessment and direct comparison of the body surface heating in the different mouse models, providing an advantage over other methods for assessing body temperature that would be limited by the accuracy and concordance of two separate temperature probes.

### Quantification and statistical analysis

#### Statistical analysis

All data points are shown as mean ± Standard Error of Means (SEM). *p* values less than 0.05 were considered statistically significant. Student’s *t* tests were used to compare two groups, while 1- or two-way ANOVA followed by Turkey–Kramer post hoc analysis was used to compare three or more groups. Statistical analyses were performed using GraphPad prism 10.0. Ink (GraphPad software). ANCOVA was utilized to correct energy expenditure data for body mass using SPSS.
