# Supplementary material for: Diet-dependent modulation of energy balance by CB1 signaling in peripheral sensory neurons
Source: iScience. 2025 Jul 15;28(8):113124. doi: 10.1016/j.isci.2025.113124 (PMC12329259; doi:10.1016/j.isci.2025.113124)
Supplement: Document S1. Figures S1–S9 [file mmc1.pdf]

iScience, Volume *28*

Supplemental information

Diet-dependent modulation of energy balance by CB1 signaling in peripheral sensory neurons

Benjamin Linden, Hussein Herz, Mohammad Jarrah, Dana Tasabehji, Sanaz Saleh, Aviva Fraer, Patrick Clark, Yuanchao Ye, Yi Chu, Zeina Al-Khalil, Donald A. Morgan, Zhiyong Zhu, Carlos M. Castorena, Leonid Zingman, Kamal Rahmouni, and Mohamad Mokadem

Supplemental Figures:

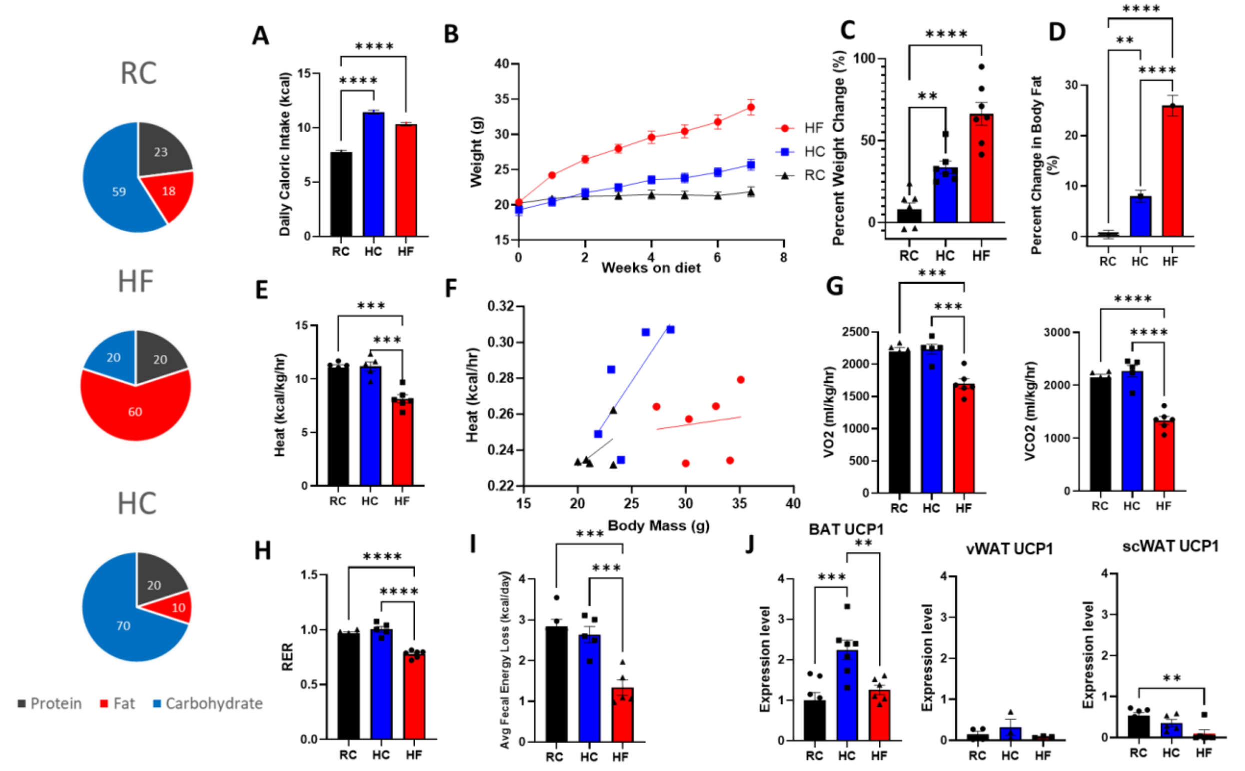

Supplemental Figure 1. Differential Effects of Several Diet Compositions on Variable Elements and Measures of Energy Balance. (**A**) Average daily caloric intake measured in kcal; (**B**) Weight Change over time; (**C**) Change in total body weight from baseline (%); (D) Change in body fat composition from baseline (%); (**E**) Average 24-hour heat production measured as kcal/kg/hr; (**F**) 24 hr Avg. heat production in (Kcal/hr) vs weights in (g); (**G**) $VO_2$and VCO_2 expressed in (mL/Kg/hr) ; (**H**) Ratio Exchange Rate (RER); (**I**) Average fecal caloric loss expressed in (Kcal/day) and, (**J**) UCP1 mRNA expression within brown adipose tissue (BAT,) visceral white adipose tissue (vWAT) and subcutaneous white adipose tissue (scWAT) in C57BL/6J male mice fed regular chow (RC), High carbohydrates/High Sucrose (HC), and High Fat (HF) diet [composition noted on the left panel] for 12 weeks using indirect calorimetry in free-moving animals. n=5-7. One-way ANOVA was used to compare the means of each diet. All results are presented as mean ± SEM (error bars). Statistical significances are denoted with asterisks as follows: *, $p \leq 0.05$; **, $p \leq 0.01$; ***, $p \leq 0.001$; ****, $p \leq 0.0001$.

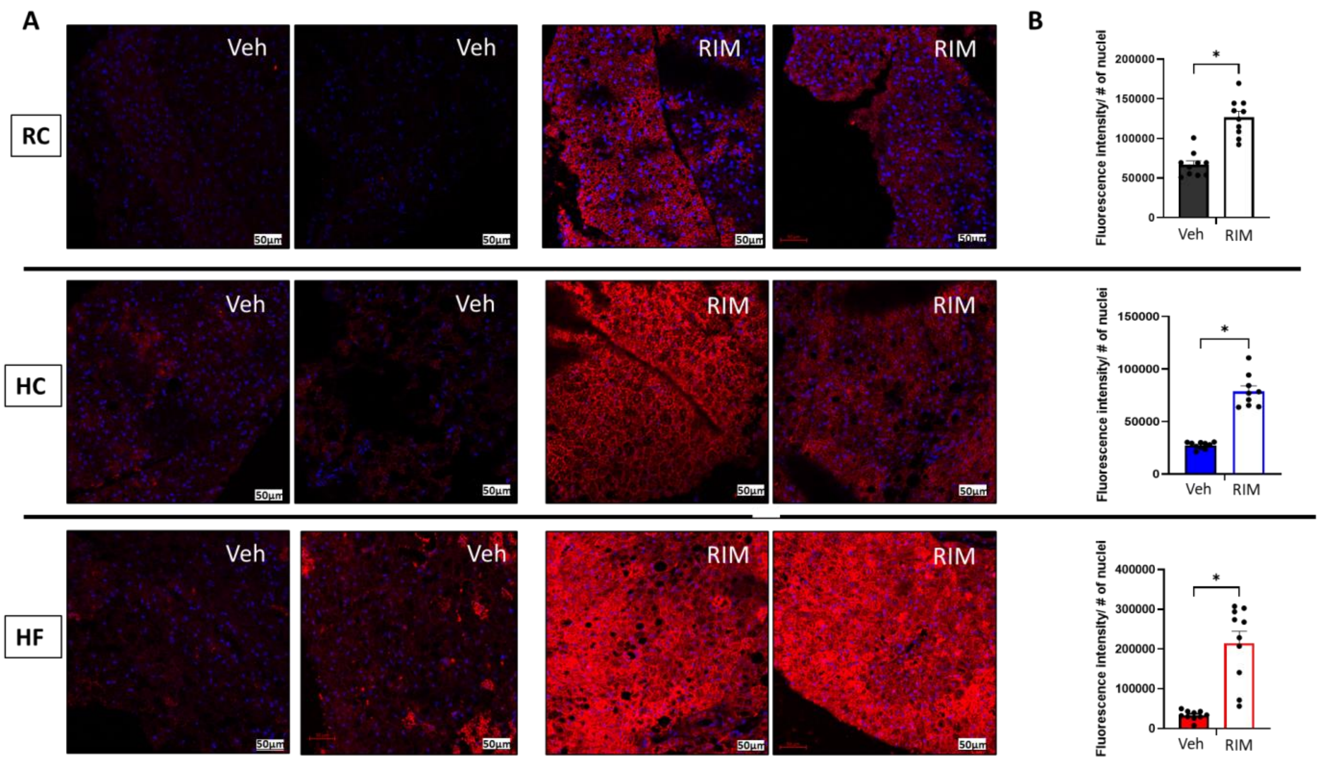

Supplemental Figure 2. UCP1 Expression in BAT. (**A**) UCP1 immuno-fluorescent (IF) staining in BAT of C57Bl/6J mice treated with vehicle vs Rimonabant for 3 weeks across 3 distinct diet groups (regular chow= RC, high-carbohydrate=HC, and high-fat=HF diets), 20x magnification. Scale bar=50 µm. .(**B**) UCP1 protein quantification in BAT tissue of vehicle vs Rimonabant-treated mice across all diets, assessed by fluorescent intensity signal. n=7-9. Student *t-test* was used to compare the means of each diet intervention. Statistical significances are denoted **p* ≤ 0.05.

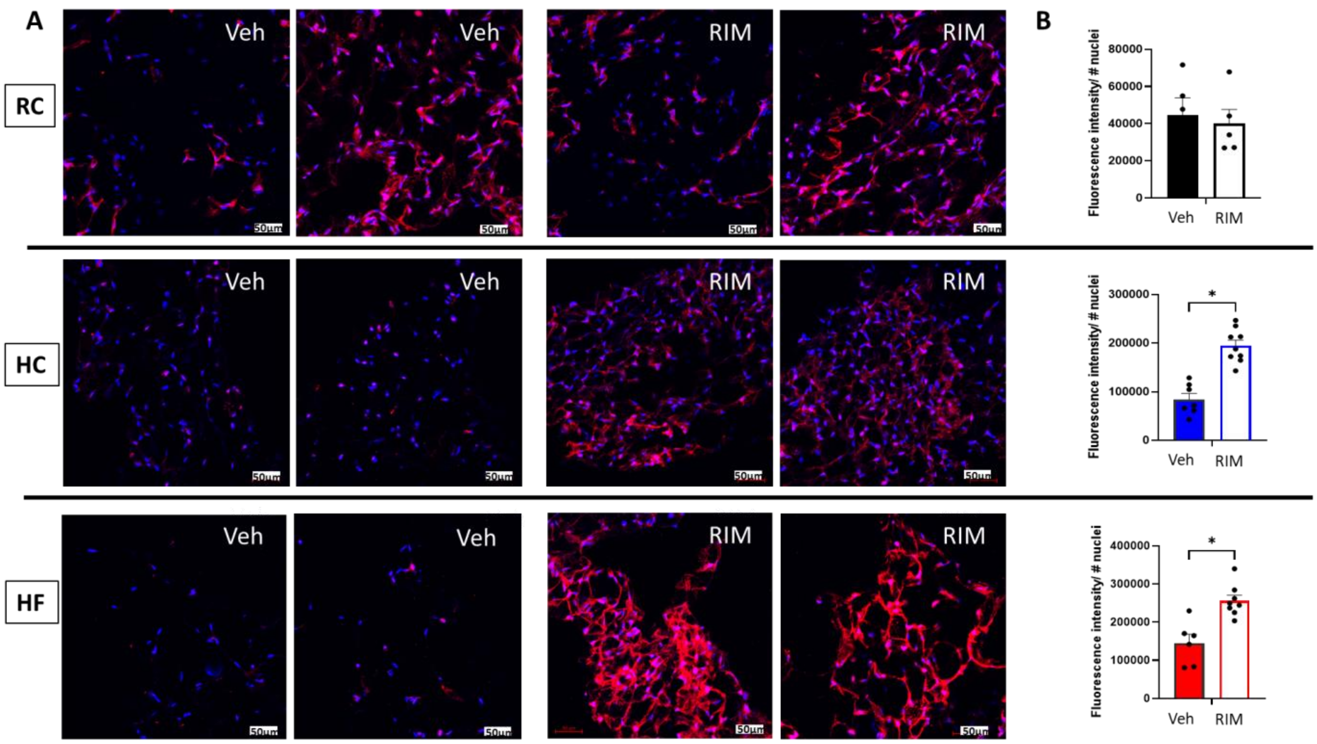

Supplemental Figure 3. UCP1 Expression in vWAT. (**A**) UCP1 immuno-fluorescent (IF) staining in vWAT of C57Bl/6J mice treated with vehicle vs Rimonabant for 3 weeks across diet groups (regular chow= RC, high-carbohydrate=HC, and high-fat=HF diets), 40x magnification. Scale bar= 50 µm. (**B**) UCP1 protein quantification in vWAT tissue of vehicle vs Rimonabant treated mice across all diets, assessed by fluorescent intensity signal. n=5-8. Student *t-test* was used to compare the means of each diet intervention. Statistical significances are denoted $*p \leq 0.05$.

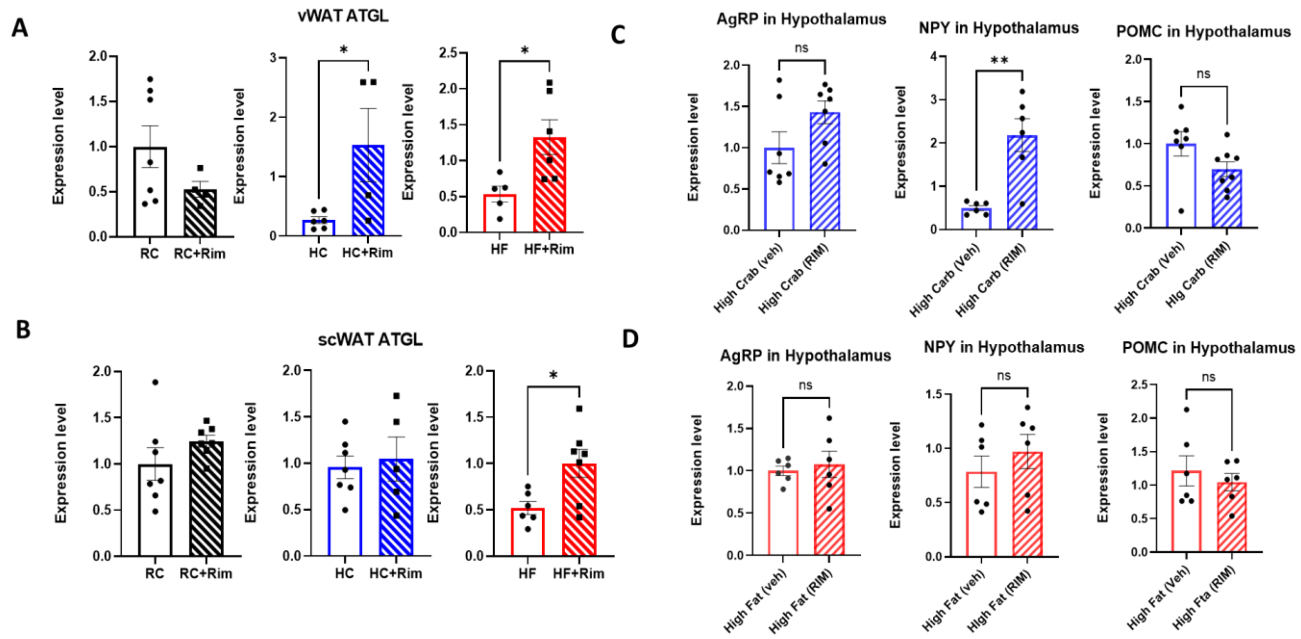

Supplemental Figure 4. mRNA expression of (**A**) ATGL in visceral white adipose tissue (vWAT) and (**B**) ATGL in subcutaneous WAT (scWAT) of C57BL/6J male mice placed on RC, HC, and HF diets and given vehicle vs Rimonabant for 21 days, and Agouti-related peptide (AgRP), Neuropeptide Y (NPY), and proopiomelanocortin (POMC) in the hypothalamus of C57BL/6J male mice after 7-day treatment of vehicle vs Rimonabant while on (**C**) HC diet and (**D**) HF diet. All results are presented as mean ± SEM (error bars). n=5-7. Student t-test was used to compare the means of each diet intervention. Statistical significances are denoted with asterisks as follows: *p ≤ 0.05; **p ≤ 0.01.

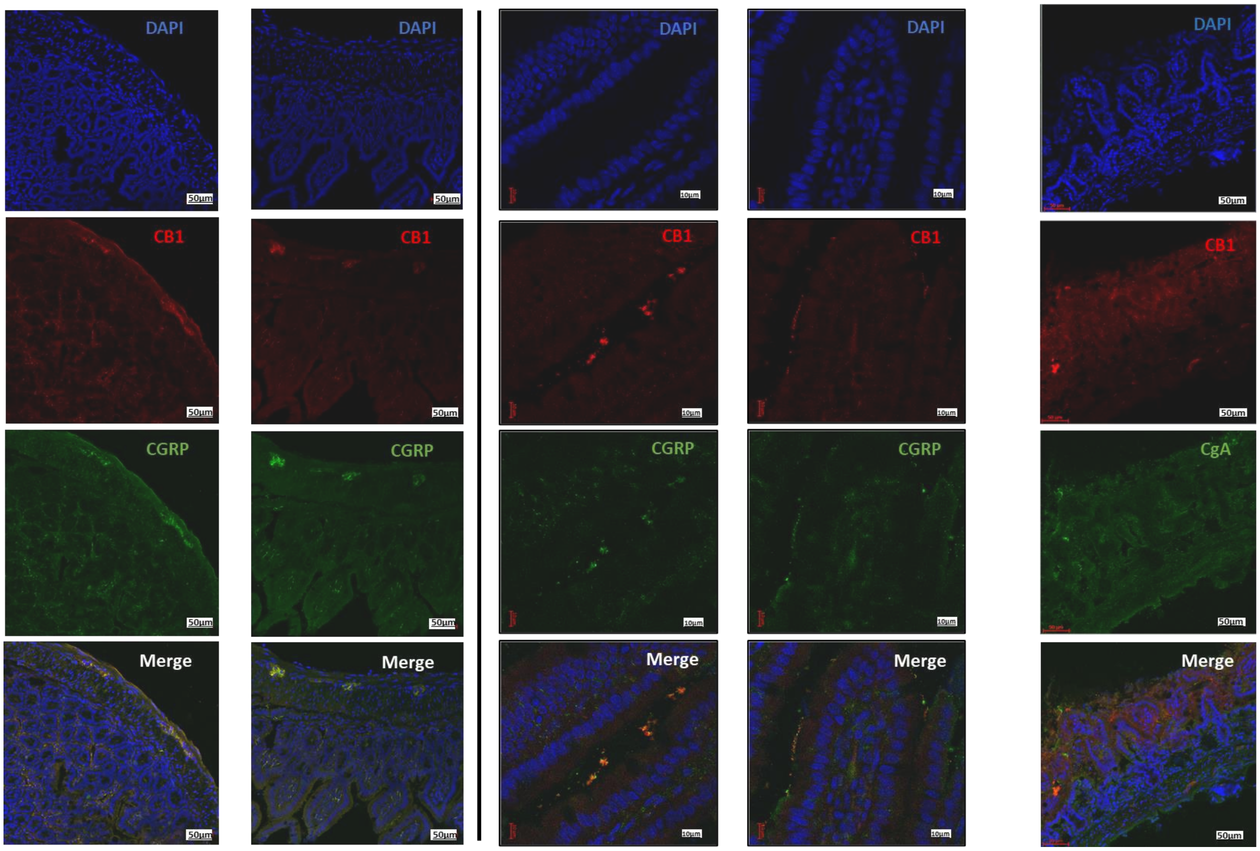

Supplemental Figure 5. Co-localization of CB1 with markers of sensory/afferent neurons (calcitonin gene-related peptide [CGRP]) and markers of enteroendocrine cells (chromogranin-A [CgA]) within the mouse's small intestine. (**A**) Co-immunostaining of CB1 with CGRP (Scale bar= 50 µm with 20X magnification, left panel and 40X magnification with vertical scale bar= 10 µm, right panel) or (**B**) CgA (right panel 20X magnification and scale bar= 50 µm) within the small intestine of HF diet-induced obese male C57BL/6J mice (CB1= Red, CGRP and CgA = Green, DAPI= Blue). n=4-5 mice.

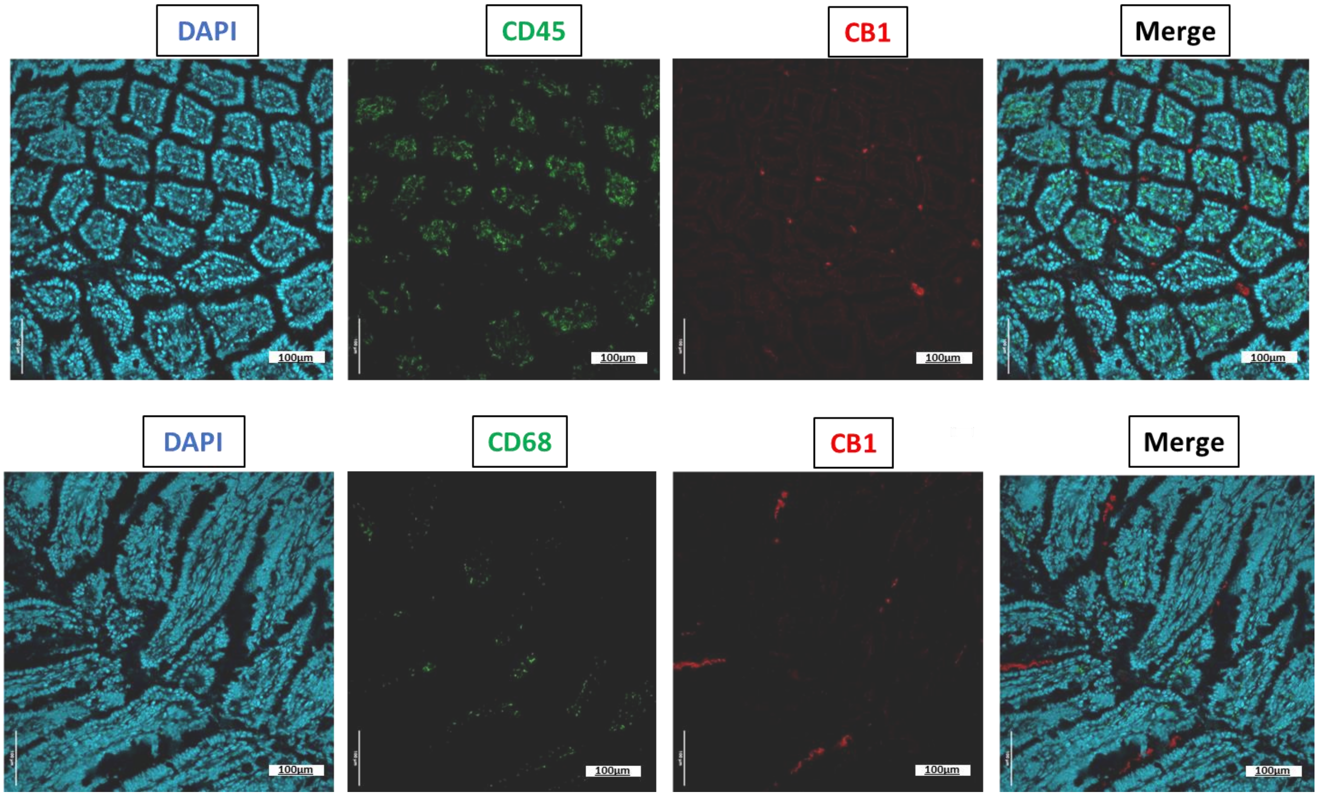

Supplemental Figure 6. Intestinal localization of CB1 [in Red] and DAPI [in Blue] with CD45 (marker for leukocytes), and CD68 (marker for macrophages/monocyte) [all in Green] using immunostaining. n=4-5.Scale bar= 100 μm.

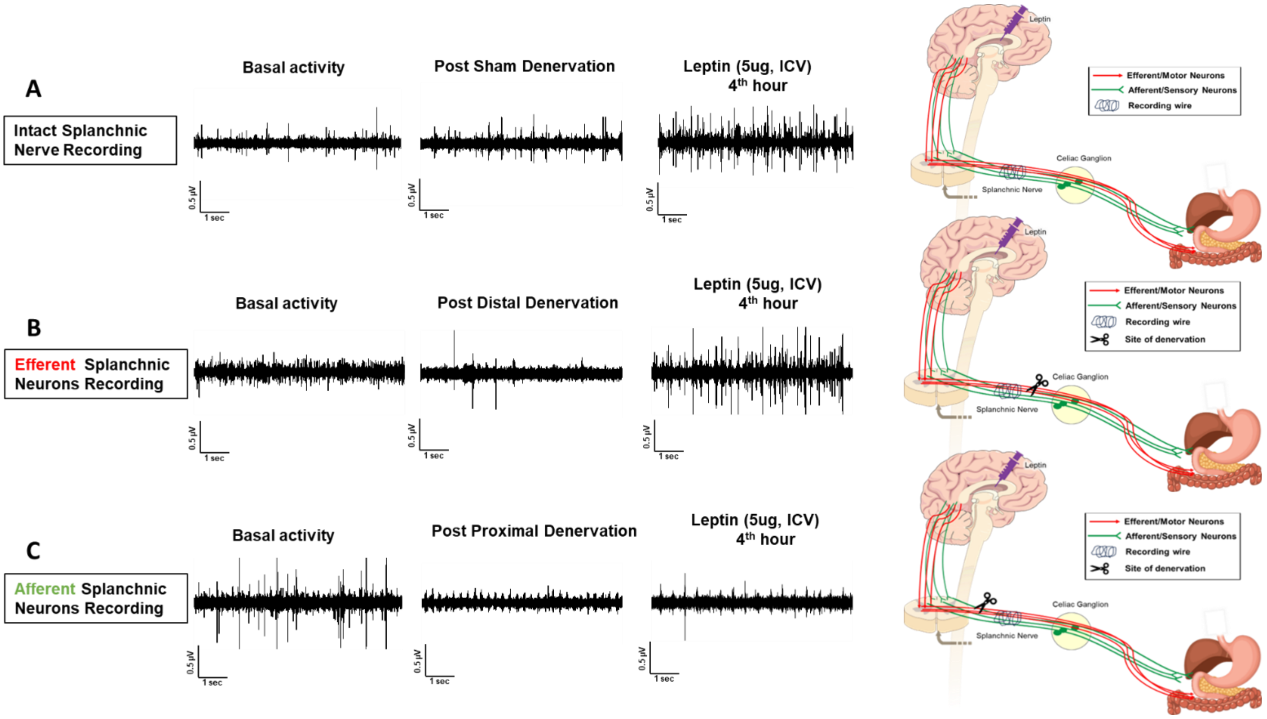

Supplemental Figure 7. ***In vivo* direct recording** of (**A**) whole or Intact splanchnic nerve (lesser branch), (**B**) motor of efferent splanchnic neurons, (**C**) sensory or afferent splanchnic neurons at baseline (*left-sided neurogram*), after sham/distal/ proximal denervation (*middle neurogram*), and 4 hours post intracerebroventricular (ICV) injection of 5µg of leptin (*right-sided neurogram*) in C57BL/6J male mice (n=3). The schematic image at the side of each intervention displays the anatomical positioning of the recording wire in relation to the denervation site.

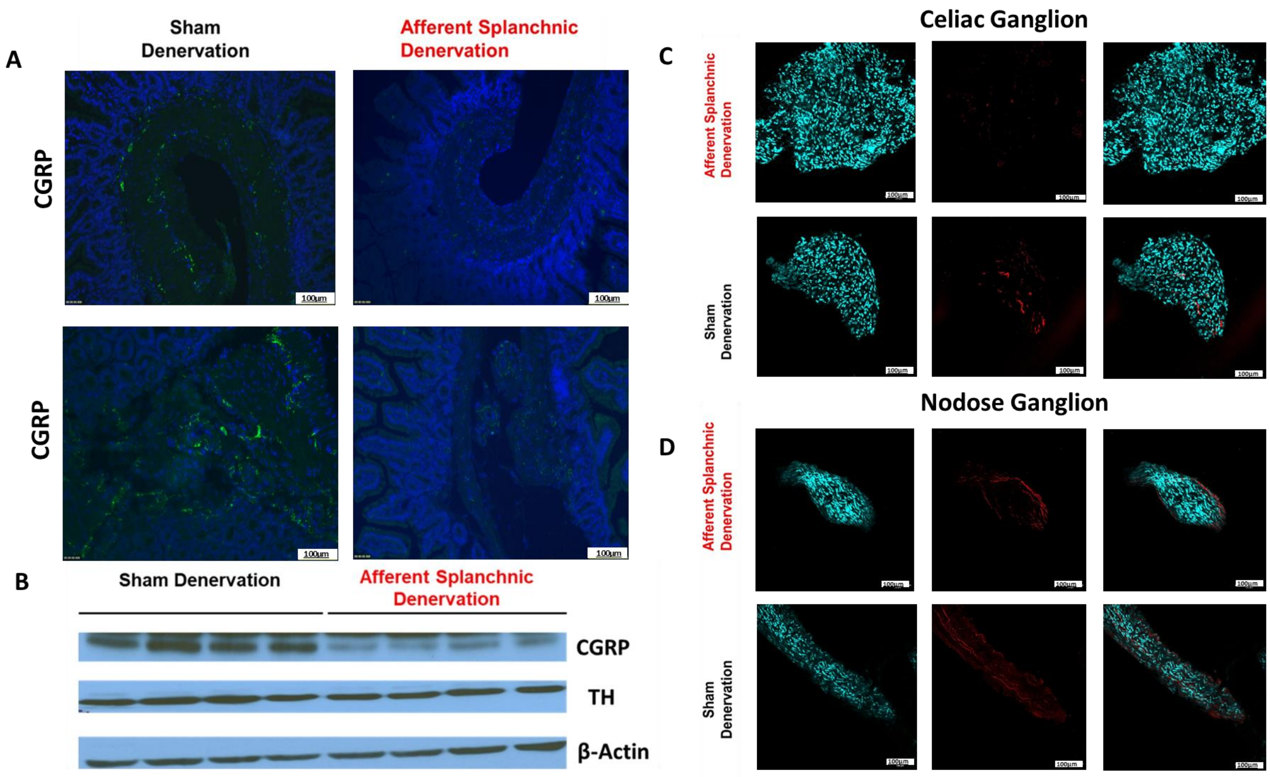

Supplemental Figure 8. A Mouse Model of Selective Afferent Splanchnic Denervation. Selective afferent splanchnic denervation was successful after delicately applying 0.2% capsaicin solution over the celiac ganglia and the pre-synaptic branches of the lesser splanchnic nerves. (**A**) Immunostaining for CGRP [in Green] in the small intestine (jejunum) of sham-denervated vs afferent splanchnic-denervated mice. (**B**) CGRP and TH expression by western blot within the small intestine (jejunum) shows selective sensory denervation with no effects on sympathetic motor fibers. Immunofluorescent (IF) staining for CB1 (in Red) and DAPI (in Blue) within (**C**) celiac ganglia [upper panel] and (**D**) nodose ganglia [lower panel] of sham-denervated vs afferent splanchnic-denervated mice. Scale bar, 100 µm.

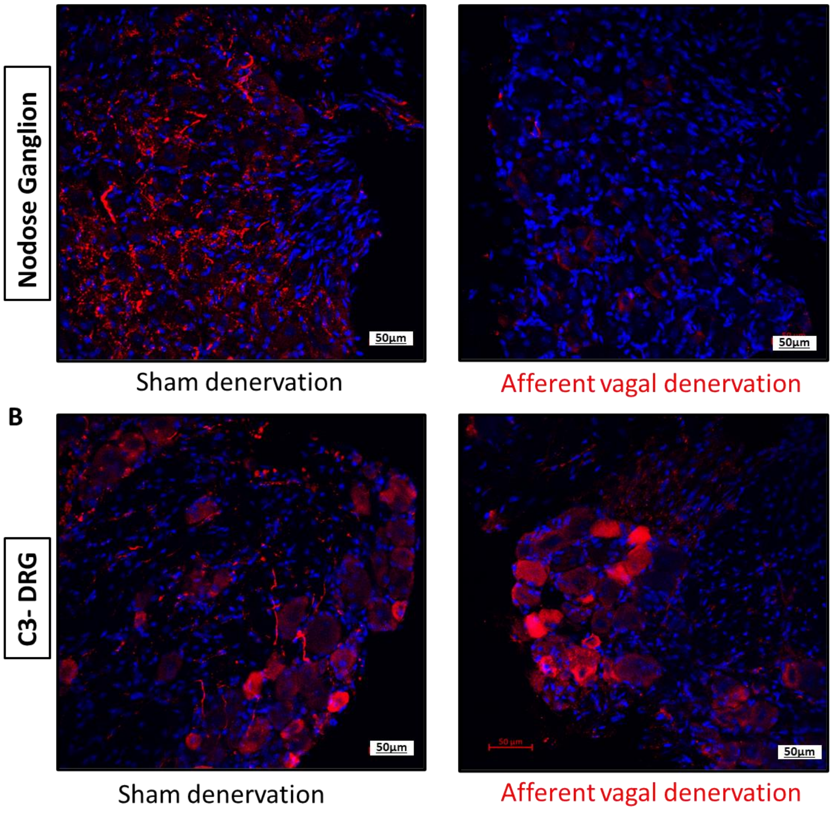

Supplemental Figure 9. A Mouse Model of Selective Afferent Vagal Denervation. Selective afferent vagal denervation was successful after delicately applying 0.4% capsaicin vs oil solution (as a vehicle) around the left (ventral) and right (dorsal) para-esophageal vagal branches. Immunostaining for CGRP in (**A**) nodose ganglia (NG) and (**B**) C3 cervical dorsal root ganglia (DRG) of C57BL/6J male mice 7 days after sham-denervation (Left) vs selective afferent vagal denervation (right). Immunofluorescent (IF) staining for CGRP is in (Red) and DAPI is in (Blue). Scale bar, 50 µm.
